# Dual Targeting of CX_3_CR1 and PARP in Models of High-Grade Serous Ovarian Carcinoma

**DOI:** 10.3390/cancers16223728

**Published:** 2024-11-05

**Authors:** Jia Xie, Maria V. Barbolina

**Affiliations:** Department of Pharmaceutical Sciences, College of Pharmacy, The University of Illinois at Chicago, 833 S Wood Str., Chicago, IL 60612, USA; athena665171@gmail.com

**Keywords:** high-grade serous ovarian cancer, fractalkine receptor, poly(ADP)-ribose inhibitor, combination treatment

## Abstract

Background/Objectives: Clinical use of poly(ADP-ribose) polymerase inhibitors (PARPis) against metastatic high-grade serous ovarian carcinoma (HGSOC) is limited to cases with deficient a homologous recombination (HR). Our objective was to determine whether the impairment of the fractalkine receptor (CX_3_CR1) could sensitize HR-proficient cases to PARPis. Methods: The efficacy of a dual drug combination, including AZD8797, an inhibitor of CX_3_CR1, and several PARPis was examined using cell lines and xenograft models. Results: The effectiveness of PARPis and AZD8797 drug combinations ranged from additive to strongly synergistic. Olaparib was synergistic with AZD8797 in OVCAR-4, Caov-3, and OHSAHO. Niraparib and AZD8797 produced synergy in OVCAR-4 and ES2. Rucaparib and AZD8797 were strongly synergistic in Caov-3 and OVSAHO. Veliparib was strongly synergistic with AZD8797 in OVCAR-4 and Caov-3. Notably, a combination of veliparib and AZD8797 produced a strong synergistic effect in a xenograft model. Conclusions: While the exact mechanisms determining the nature of the PARPis and AZD8797 interaction remain to be uncovered, our data indicate that, in a subset of models, selected PARPis strongly synergize with the inhibition of CX_3_CR1, suggesting a potential therapeutic opportunity.

## Simple Summary

About half of all cases of high-grade serous ovarian carcinoma is deficient in the homologous recombination pathway, while the other half is proficient, and the latter patient cohort cannot benefit from targeted therapies containing inhibitors of poly(ADP-ribose) polymerase. Our previous study indicated that impairment of CX_3_C motif chemokine receptor 1, or the fractalkine receptor, renders cells deficient for homologous recombination. The goal of this study was to investigate the efficacy of the co-targeting fractalkine receptor and poly(ADP-ribose) polymerase in models of high-grade serous ovarian cancer. We found that combinations of inhibitors of poly(ADP-ribose) polymerase and an inhibitor of the fractalkine receptor produced a wide range of responses, ranging from additive to strongly synergistic. These data indicate that the dual targeting of the fractalkine receptor and poly(ADP-ribose) polymerase can offer a new therapeutic approach and potentially extend the use of poly(ADP-ribose) polymerase inhibitors to most cases of high-grade serous carcinoma, irrespective of their homologous recombination capacity.

## 1. Introduction

For the past several decades, epithelial ovarian carcinoma (EOC) has remained the fifth leading cause of female cancer mortality and the deadliest gynecologic malignancy [1]. EOC is a complex disease that originates from precursor cells within tissues of the ovary, fallopian tubes, and the uterus, and the high-grade serous histotype (HGSOC) of EOC is the most predominant and the deadliest [2,3,4]. Unfortunately, reliable and affordable early screening approaches for this largely sporadic malignancy are lacking [5]. As a result, most patients present when metastasis has already occurred; thus, treatment of the metastatic disease becomes a critical clinical challenge.

Ovarian carcinoma typically disseminates by detaching from its primary site and lodging onto the serosa of the organs and tissues of the peritoneal cavity, which eventually causes patients’ death via malnutrition and bowel obstruction [6]. Patients with metastatic ovarian carcinoma typically undergo debulking surgery and first-line cytotoxic chemotherapy with a microtubule-stabilizing taxane and a DNA damage-inducing platinum agent [7]. However, intrinsic and acquired resistance to these agents limits their efficacy, and recurrent metastatic disease ultimately becomes chemotherapy-resistant, leaving patients with no viable treatment options [8].

The Cancer Genome Atlas project has reported that about half of cases of HGSOC carries defects in the homologous recombination (HR) pathway [9]. HR is required to repair lethal double-strand (ds) DNA breaks, which occur during DNA replication and exposure to exogenous stimuli, including X-ray radiation and platinum-containing chemotherapy. Deficiencies in HR have previously been associated with increased sensitivity to PARPis [10], because functional PARPs are required for the repair of dsDNA damage by HR, as well as non-homologous end joining (NHEJ) and alternative NHEJ [11]. As BRCA-deficient cells depend on PARPs to repair single-strand DNA damage, PARPis can induce synthetic lethality in cells with impaired BRCA1/2, which occurs in 5–10% of patients with HGSOC. Cells deficient in other genes related to HR DNA repair mechanisms are also sensitive to PARPi treatment [12]. Importantly, PARPis are promising new targeted therapies that have shown efficacy in several clinical trials. A clinically significant increase in progression-free survival (PFS) prompted the FDA’s approval of olaparib and niraparib as frontline maintenance therapy for BRCA-mutated and all cases of ovarian cancers, respectively [13,14]. Additionally, olaparib, niraparib, and rucaparib are FDA-approved to treat recurrent ovarian, fallopian tube, and primary peritoneal cancers [15,16,17]. While veliparib is not yet approved to treat ovarian cancer, a statistically significant increase in PFS was reported in patients with BRCA1/2-mutated diseases who were treated with the “veliparib throughout” regimen, consisting of carboplatin and paclitaxel plus veliparib followed by veliparib maintenance [18].

However, the remaining half of the HGSOC cases is known to be proficient in HR. For those cases, PARPis are largely ineffective. Hence, impairing HR in these tumors could offer a therapeutic opportunity to use PARPis. Clinical data indicate that the induction of HRD with targeted agents could expand the application of PARPis to HR-proficient tumors [19,20]. For example, several clinical trials reported a statistically significant increase in PFS in patients treated with anti-angiogenic drugs in combination with PARPis versus PARPis alone [21]. The underlying mechanism is thought to rely on blocking VEGF-induced NHEJ and HR [22].

We previously uncovered the role of C-X_3_-C motif chemokine receptor 1 (CX_3_CR1) in DNA damage recognition and repair. Our studies indicate that the impairment of CX_3_CR1 can synergize with dsDNA-damaging agents to eliminate ovarian cancer cells [23]. We demonstrated that the downregulation of CX_3_CR1, together with the administration of DNA-damaging agents, such as X-ray radiation and cisplatin, led to a significant decrease in the activation of ataxia–telangiectasia mutated (ATM) kinase and resulted in a subsequent significant increase in dsDNA damage compared to either of the stimuli (CX_3_CR1 downregulation or DNA-damaging agent) alone [23]. Expanding on these previous studies, we hypothesized that the loss of CX_3_CR1 may render ovarian carcinoma cells vulnerable to PARPis as well. To test this premise, we examined whether PARPis could synergize with the loss of CX_3_CR1 in (a) reducing clone formation in HGSOC cell lines and (b) reducing tumor growth in xenograft mouse models.

## 2. Materials and Methods

### 2.1. Cell Lines

Human-derived epithelial ovarian carcinoma cell line OVCAR-4 was obtained from the NCI Tumor Cell Repository (Detrick, Fort Detrick, MD, USA). Human ovarian carcinoma cell line of serous histotype OVSAHO was obtained from the Japanese Collection of Research Bioresources Cell Bank (Osaka, Japan). Human-derived ovarian carcinoma cell lines Caov-3 and ES-2 were obtained from Dr. M.S. Stack (University of Notre Dame, Notre Dame, IN, USA). All cell lines were cultured, as suggested by the manufacturers, for no longer than 15 consecutive passages and routinely assessed by cell morphology, average the doubling time, and a short tandem repeat analysis that indicated a 100% match of cell line-specific DNA loci to the tested samples. The cell lines were free from contamination by *Mycoplasma fermentans*, as determined using The LookOut Mycoplasma PCR Detection kit (Sigma-Aldrich, St. Louis, MO, USA).

### 2.2. Mice

Female, 6–8-week-old, athymic, nude—FOXN1^NU^ mice were obtained from Envigo (Indianapolis, IN, USA). All experimental procedures were performed according to the Institutional Animal Care and Use Committee protocol approved by the Animal Care Committee of UIC. Animals were fed ad libitum and maintained in the Association for Assessment and Accreditation of Laboratory Animal Care International-approved facilities on a 12 h light–12 h dark cycle.

### 2.3. Antibodies and Reagents

Rabbit polyclonal anti-human ɣH2AX, as well as control siRNAs and a pool of CX_3_CR1-specific siRNAs, were obtained from Santa Cruz Biotechnology (Dallas, TX, USA). Mouse monoclonal anti-human β1-actin was obtained from Invitrogen (Waltham, MA, USA). Rabbit polyclonal anti-human CX_3_CR1 antibodies (ab8021) were obtained from Abcam (Waltham, MA, USA). Goat anti-rabbit IgG DyLight488 antibodies were purchased from Vector Laboratories (Newark, CA, USA). AZD8797, olaparib, niraparib, rucaparib, and veliparib were obtained from MedChemExpress (Monmouth Junction, NJ, USA). Crystal violet was obtained from Sigma–Aldrich (St. Louis, MO, USA). Hydroxy-beta-cyclodextrin was obtained from Acros Organics (Waltham, MA, USA). Dharmafect reagent 1 was obtained from GE Dharmacon (Marlborough, MA, USA).

### 2.4. Radiation Treatments

Transfected and control cells were plated in tissue culture-treated 6-well plates and subjected to a single dose of X-ray radiation at 0.8 gray/min using a linear accelerator (UIC Radiation Oncology) 3 days following transfection. The parameters of the linear quadratic model were used to compare radiosensitivity, provided that the data were statistically significant, using the one-way ANOVA test. The radiation enhancement factor (REF) and dose modifying factor (DMF) were used to compare radiosensitivity between the control and the treatment groups. The REF is defined by the surviving fraction of the control group compared to that of the treatment groups at 2 gray (SF2_control_/SF2_treatment_). The DMF is defined as the dose required to kill 90% of the population in the control group compared to that in the treatment group (DMF10_control_/DMF10_treatment_). When REF2 and DMF10 were larger than 1.1, the treatment group was considered radiosensitized.

### 2.5. Clonogenic Assay

After radiation or drug treatment, 1–5% of the cells were replated on 100 mm^2^ diameter tissue culture plates and allowed to grow for up to 2 weeks until visible colonies of more than 50 cells formed. Cells were fixed with 4% paraformaldehyde (PFA) and stained with 0.05% crystal violet solution. Colonies were quantified and used to create survival curves. The surviving fraction of cells was obtained by detecting OD450 values and plotting using SigmaPlot 12.5 software (SigmaPlot, London, UK). Both quantification methods produced similar results.

In drug treatment experiments, cells were treated with drugs for 48 h, and then 1–5% of the treated cells and untreated controls were examined with a clonogenic assay until colonies formed in the untreated controls. The number of colonies containing >50 cells was enumerated and averaged from 6 independent experiments. Percent of the effect was calculated using the equation: effect = (number of colonies in control − number of colonies in the experiment)/(number of colonies in control) × 100%. The combination index (CI) was quantified using the Bliss Independence model: CI = (DrugA_effect_ + DrugB_effect_ − DrugA_effect_×DrugB_effect_)/(DrugAB_effect_), where DrugA_effect_ corresponds to the effect of AZD8797 alone, DrugB_effect_ corresponds to the effect of a PARPi alone, and DrugAB_effect_ corresponds to the effect of the drug combination [24,25]. The nature of drug interaction was determined as synergistic at CI < 0.9, additive when 0.9 ≤ CI ≤ 1.1, and antagonistic at CI > 1.1.

### 2.6. Western Blot

Procedures were conducted as described in [23,26,27]. The proteins were visualized using horseradish peroxidase-based detection and BioRad Chemidoc imager.

### 2.7. Tumor Formation and Treatments

The 10^6^ OVCAR-4 or ES-2 cells were injected intraperitoneally (i.p.) into athymic nude mice. The animals were treated, or not, with either AZD8797 or PARPis (olaparib, niraparib, and veliparib) or their combination. Hydroxypropil-beta-cyclodextrin (hpbcd) was dissolved in sterile water at a 45% (weight/volume) final concentration, sterilized by passing it through a 0.22 micron filter, and administered as vehicle control treatment by oral gavage using feeding tubes at a volume of 200 µL. AZD8797 was dissolved at 45% (*w*/*v*) hpbcd at 6.25 mg/mL, sterilized by passing it through a 0.22 micron filter, and delivered by oral gavage using feeding tubes at a volume of 100 µL. Olaparib, niraparib, and veliparib were dissolved at 45% (*w*/*v*) hpbcd at 2.5, 6.25, and 3.33 mg/mL, respectively; sterilized by being passed through a 0.22 micron filter; and delivered by oral gavage using feeding tubes at a volume of 100 µL. ES-2-bearing animals were monitored three times weekly until they reached humane endpoints and sacrificed. OVCAR-4-bearing animals were monitored 3 times weekly and were sacrificed 3 months after tumor inoculation. Tumors were excised, weighed, fixed in paraformaldehyde, and paraffin-preserved, as described before [23,26,28,29].

### 2.8. Immunofluorescence Staining and Imaging

The AxioVert Zeiss fluorescence microscope was used as detailed in [23,26,28,29]. ɣH2AX antibodies were used at a 1:250 dilution overnight at 4 °C. Rabbit DyLight488 secondary antibodies were used at a 1:500 dilution for 1 h at room temperature. DAPI was used to visualize nuclear DNA.

### 2.9. Statistics

Comparisons between two datasets with a normal distribution were conducted using Student’s *t*-test and Microsoft Excel 2016 software. The Mann–Whitney U test was used to compare two datasets with abnormal distributions. Differences were considered statistically significant at *p* < 0.05. Two-way ANOVA was used to analyze datasets with more than two groups. The Kaplan–Meier estimator and GraphPad PRISM 8 software were used to analyze survival.

## 3. Results

### 3.1. Impairment of CX_3_CR1 Potentiates’ Efficacy of PARPis In Vitro

#### 3.1.1. Downregulation of CX_3_CR1 with Specific siRNAs and X-Ray Radiation Significantly Increases the Effectiveness of Olaparib Compared to X-Ray Radiation Alone

**Figure 1:**
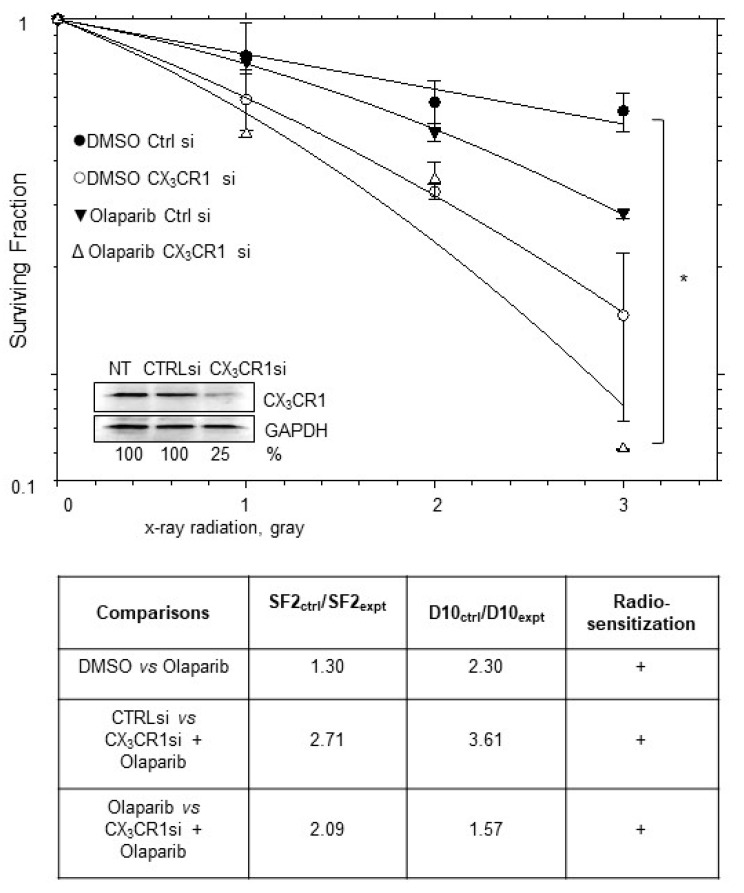
Downregulation of CX_3_CR1 synergizes with X-ray radiation to reduce clone formation. Caov-3 was transiently transfected with either CX_3_CR1-specific (designated “CX_3_CR1si”) or control (designated “Ctrl si”) si RNAs or the vehicle (designated “NT”) and subjected to 0, 1, 2, or 3 gray X-ray radiation, 10 µM olaparib, or DMSO, as indicated, on the 3rd day following transfection. CX_3_CR1 expression was determined using Western blot; GAPDH was used as the loading control. Normalized expression of CX_3_CR1 was calculated using digital densitometry. Original image of Western blot can be found at supplementary file. Survival curves are the average of five independent experiments. * *p* < 0.05; two-way ANOVA test. Radiosensitization shown in the table was determined if both SF2_control_/SF2_CX3CR1_ and D10_control_/D10_CX3CR1_ were >1.1; SF2—surviving fraction at 2 gray, D10—radiation dose required to kill 90% of the cell population.

We previously reported that elevated CX_3_CR1 expression in ovarian carcinoma contributes to the dsDNA repair process and its reduction sensitizes cells to DNA-damaging therapeutic modalities, including X-ray radiation [23]. Building on these observations, we found that, while olaparib and the downregulation of CX_3_CR1 were more effective in radiosensitizing the irradiated BRCA wild-type tumor cell line Caov-3 [30], which is HR-proficient [31], all three interventions, including CX_3_CR1 downregulation, olaparib, and X-ray radiation, were significantly more effective when used together (Figure 1). These findings indicate that the impairment of CX_3_CR1 can be a potentially viable option for increasing the effectiveness of PARPis in HR-proficient HGSOC.

#### 3.1.2. Pharmacologic Inhibition of CX_3_CR1 and PARP Yields Reduced Clonogenic Ability in In Vitro Models of HGSOC

**Figure 2:**
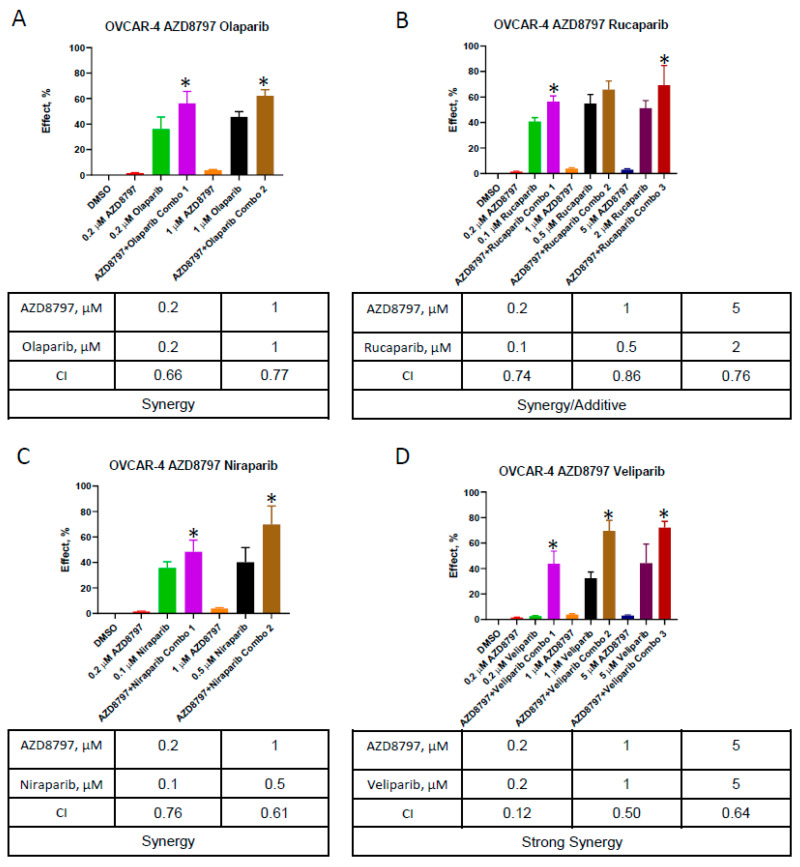
Drug interaction between AZD8797 and PARPis in OVCAR-4. OVCAR-4 cells were treated with olaparib (**A**), rucaparib (**B**), niraparib (**C**), and veliparib (**D**) at indicated concentrations for 48 h and examined with a clonogenic assay. Percent of the effect was calculated using the equation: effect = (number of colonies in control − number of colonies in the experiment)/(number of colonies in control) × 100%. An average of six independent experiments is shown. The data between two groups (individual drugs vs combinations) were analyzed using the Mann–Whitney U test; * *p* < 0.05. The tables summarize the data for all tested combinations.

While X-ray radiation is a reliable and robust tool to induce dsDNA breaks and mimic HR deficiency, it is less useful in clinical practice due to its systemic side effects on normal tissue. Based on our data indicating that the impairment of CX_3_CR1 can result in the reduced recognition and repair of dsDNA, we wished to examine if this approach could effectively sensitize the HR-proficient models of HGSOC to PARPis. To systematically explore the role of CX_3_CR1 in increasing the efficacy of PARPis, we selected several cell culture models with which we investigated whether the impairment of CX_3_CR1 affects PARPi treatment outcomes. OVCAR-4 and Caov-3 are BRCA wild-type models of HGSOC, and OVSAHO is an HGSOC model containing a deletion of BRCA2 [30,32]. ES2 is a BRCA wild-type cell line that was originally described as a model of a clear cell histotype of ovarian cancer, although it was found to display features of the HGSOC histotype, such as mutated TP53 [33]. The Cancer Genome Atlas study demonstrated that CCNE1 amplification, a feature of OVCAR-4 [34], defines HR-proficient tumors [9]. Caov-3 was shown to be HR-proficient using functional read-out assays [31]. ES2 was found to be HR-proficient by examining the RAD51 foci formation after irradiation [35]. Additionally, the pharmacological inhibition of CX_3_CR1 with AZD8797, an allosteric inhibitor of the receptor’s activation [36,37], was selected over the genomic downregulation to examine impairing CX_3_CR1 as a potentially viable clinical approach. Although our initial findings were based on using X-ray radiation as a modality to induce dsDNA breaks, in this follow-up study, we relied only on the inhibition of CX_3_CR1 to induce a state of HR deficiency to avoid possible toxic side effects arising from a combination of several therapeutic modalities, which could prevent the future clinical use of this approach. Cell lines OVCAR-4, Caov-3, OVSAHO, and ES2 were treated in the presence and absence of AZD8797, olaparib, rucaparib, niraparib, veliparib, and combinations of AZD8797 with either olaparib, or rucaparib, or niraparib, or veliparib, and then examined for their ability to proliferate after treatment in a clonogenic assay. The concentration range of the inhibitors was selected to yield not less than 50% reduction in clone formation by individual drugs to determine the effect of the combinations. We found that all tested PARPis synergized with AZD8797 to significantly reduce the clonogenic ability of OVCAR-4, and a combination of AZD8797 with veliparib induced the strongest levels of synergy (Figure 2).

**Figure 3:**
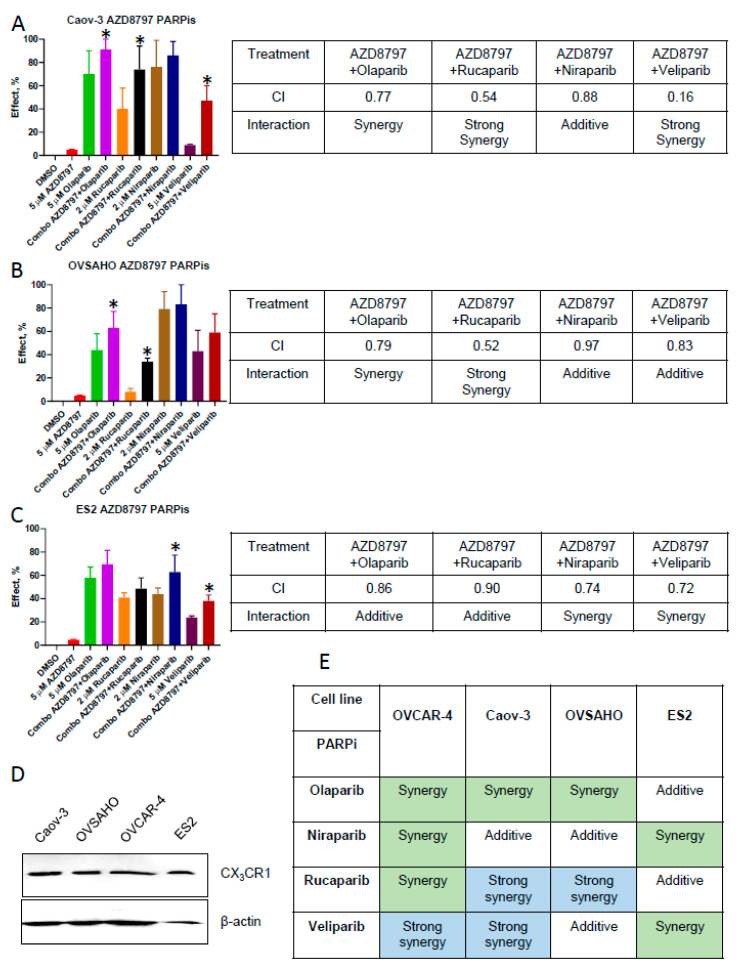
Drug interaction between AZD8797 and PARPis in HGSOC cell lines. Caov-3 (**A**), OVSAHO (**B**), and ES2 (**C**) cells were treated with drugs at the indicated concentrations for 48 h and examined with a clonogenic assay. An average of six independent experiments is shown. The data between two groups (individual drugs vs combinations) were analyzed using the Mann–Whitney U test; * *p* < 0.05. The tables summarize the data for all tested combinations. (**D**) Expression of CX_3_CR1 was examined with Western blot in all tested cell lines. Original image of Western blot can be found at supplementary file. Expression of β-actin was used as a loading control. (**E**) The table summarizes the nature of AZD8797’s and PARPis’ interaction in all tested cell lines.

In Caov-3, combinations of AZD8797 with either olaparib, rucaparib, or veliparib were synergistic in reducing the clone formation, and the strongest levels of synergy were observed with veliparib and rucaparib, and a combination of AZD8797 and niraparib had an additive effect (Figure 3A). Combinations of AZD8797 and olaparib and AZD8797 and rucaparib were synergistic in reducing clone formation in OVSAHO, with the latter displaying strong levels of synergy; however, combinations of AZD8797 and either niraparib or veliparib had an additive effect on this cell line (Figure 3B). Lastly, combinations of AZD8797 with either olaparib or rucaparib produced an additive effect on the clonogenic ability of ES2, while combinations of AZD8797 with either niraparib or veliparib were synergistic (Figure 3C). Of note, all tested cell lines expressed CX_3_CR1 (Figure 3D). In sum, we observed that combinations of AZD8797 with PARPis displayed various types of drug interactions, ranging from strongly synergistic to additive, depending on a PARPi and the cell line background (Figure 3E).

### 3.2. Impairment of CX_3_CR1 Potentiates the Efficacy of PARPis In Vivo

We wished to examine whether combined drug effects observed in vitro are carried out in the in vivo setting. To address this issue, we selected several xenograft models and different drug treatment regimens based on the tumor growth kinetics of each model and the drug interaction results from cell culture models.

**Figure 4:**
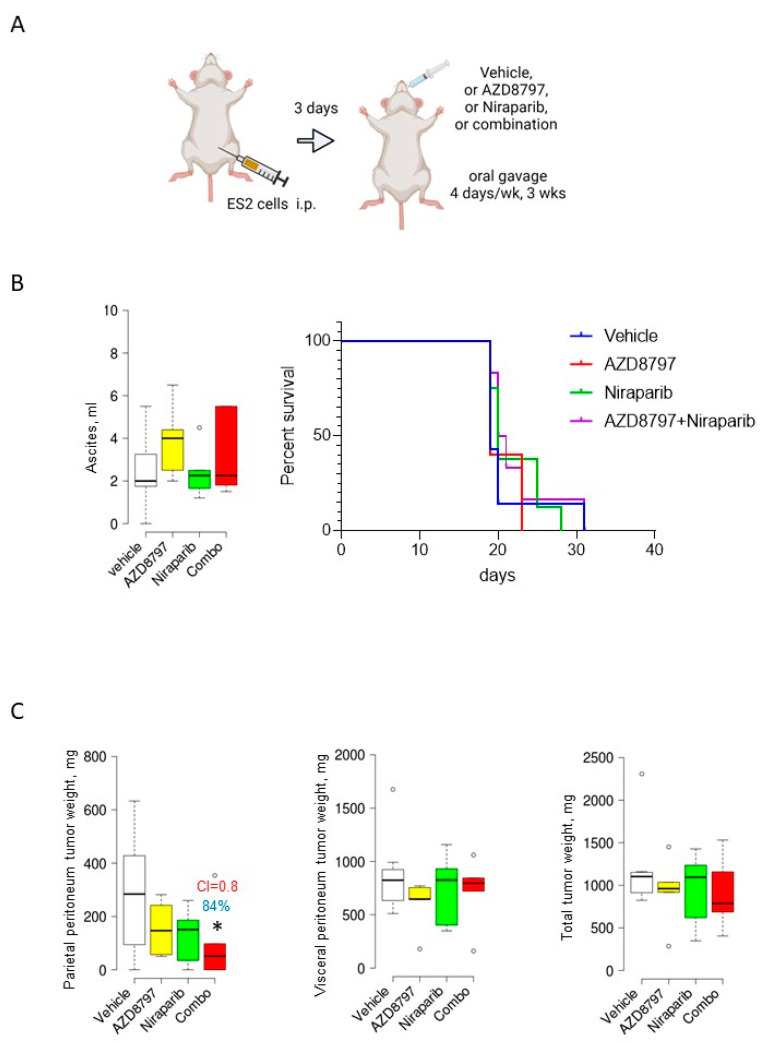
Combining niraparib and AZD8797 significantly reduces tumor weight at parietal peritoneum sites in the ES2 xenograft model. (**A**) The scheme represents an experimental design where 10^6^ ES2 cells were i.p. injected into athymic nude mice (*n* = 10/group), allowed to form tumors for 3 days, followed by the treatment with either AZD8797 (0.625 mg/mouse) or niraparib (0.625 mg/mouse) or their combination that was delivered by oral gavage on four consecutive days followed by three consecutive drug-free days for three weeks and sacrificed. The images were created in BioRender. (**B**) At the time of sacrifice, the ascites were collected, measured, plotted with BoxPlotR software (chemgrid.org), and analyzed using the Mann–Whitney U test. The overall survival was plotted using GraphPad Prism 8 software and analyzed using the log-rank (Mantel–Cox) test. (**C**) The tumor burden at parietal and visceral peritoneum sites for each animal in all groups was found by weighing the excised tumor specimens, then plotted as box plots using BoxPlotR software and statistically analyzed with the Mann–Whitney U test between vehicle (hpbcd)-treated and drug-treated groups. * *p* < 0.05. The combination index (CI) was quantified using the Bliss Independence model. Percent of tumor reduction is indicated on the graphs.

We selected an ES2/athymic nude mouse model to examine the effects of AZD8797 and niraparib, which were weakly synergistic in our in vitro experiments (Figure 3C). There are several advantages to using an ES2 xenograft model, including its aggressive nature and a propensity to develop wide-spread intra-abdominal tumors involving both the parietal and visceral peritoneum, and large volumes of ascites, commonly observed in the majority of HGSOC patients [38]. ES2 samples were i.p. inoculated into mouse abdomens and allowed to lodge for 3 days, followed by treatment with either AZD8797 or niraparib, or their combination via oral gavage. Treatments continued for 3 weeks and were delivered on four consecutive days, followed by a three-day drug-free period each week (Figure 4A). Animals were monitored until they reached humane endpoints and sacrificed. The development of ascites was the terminal factor. We found that neither the individual drugs nor their combination affected ascites’ formation and the overall survival (Figure 4B). However, when tumors were dissected and analyzed, the group that was treated with the AZD8797 and niraparib drug combination had a significant reduction in tumor burden at tissues outlined by the parietal peritoneum, including the diaphragm and peritoneal wall. The combination index indicated that the combined treatment had a weakly synergistic effect (Figure 4C). Tumor lesions that formed at the sites outlined by the visceral peritoneum, including the omentum, mesentery, and serosa of the bowels, were unaffected by either drugs individually or their combination; and since the sizes of tumors formed at the visceral peritoneal surfaces were much larger than those that formed at the parietal peritoneum tissues, the total tumor burden was found to be not affected by the treatments (Figure 4C). These results indicate that the AZD8797 and niraparib combination is weakly synergistic in ES2, both in vitro and in vivo (at parietal peritoneal sites). At the same time, the volume of ascites and the size of the visceral peritoneal tumors were not affected by this treatment, indicating a putative role of other confounding factors in the microenvironment and, possibly, a suboptimal drug administration regimen.

**Figure 5:**
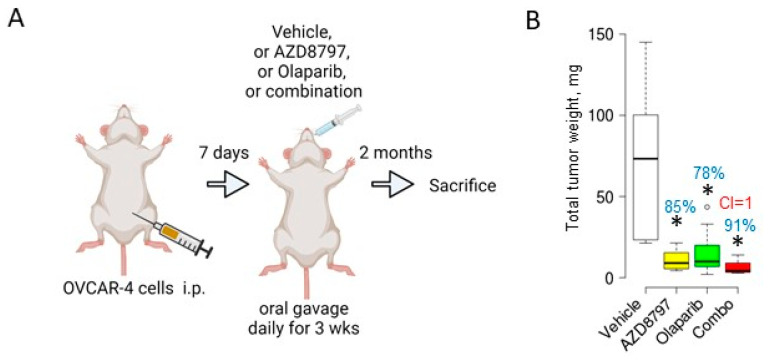
Daily administration of olaparib and AZD8797 significantly reduces tumor weight in the OVCAR-4 xenograft model. (**A**) The scheme represents an experimental design where 10^6^ OVCAR-4 cells were i.p. injected into athymic nude mice (n = 10/group), allowed to form tumors for 7 days, followed by the treatment with either AZD8797 (0.625 mg/mouse) or olaparib (0.25 mg/mouse) or their combination that was delivered by oral gavage daily for three weeks, followed by a two-month drug-free period and sacrificed. The images were created in BioRender. (**B**) At the time of sacrifice, the total tumor mass was collected, measured, and plotted with BoxPlotR software (chemgrid.org), and analyzed using the Mann–Whitney U test between the vehicle-treated and drug-treated groups. * *p* < 0.05. The combination index (CI) was quantified using the Bliss Independence model. Percent of tumor reduction in drug-treated groups in comparison to the vehicle (hpbcd)-treated control group is indicated on the graphs.

We next selected the OVCAR-4 xenograft model because this cell line carries genetic and phenotypical attributes of HGSOC. It produces little to no ascites in the in vivo xenograft setting, which allows us to eliminate this parameter from evaluating the drug effectiveness and focus only on the tumor size as the main outcome. Furthermore, OVCAR-4 develops tumor lesions primarily at the visceral peritoneum, which allows the examination of the treatment’s effectiveness specifically at these sites. Per our prior experience, this xenograft model is more indolent than the ES2 model [23,26,28,29]; therefore, inoculated cells were allowed to adhere and lodge peritoneal lesions for seven days (Figure 5A). Drug dosing was intensified because the regimen used to treat the ES2 xenograft model was ineffective at reducing tumors at visceral peritoneal sites. AZD8797, olaparib, and their combination were administered by oral gavage once daily for three weeks. After the three-week drug treatment, the animals were maintained drug-free for two months to establish the long-term effect of the treatment and to examine the durability of the response, and then sacrificed. AZD8797 and olaparib significantly and robustly reduced the tumor burden by 85% and 78%, respectively (Figure 5B), indicating their high potential effectiveness as daily monotherapies in this model. The combination of these drugs was more effective than the individual drugs and further reduced the residual tumor burden by 91%, although the nature of the drug interaction was additive. As tumors were allowed to develop only for seven days post-inoculation, it is likely that small and less-established tumor lesions were more susceptible to daily drug treatments that resulted in the high efficiency of both monotherapies that did not allow for the accurate testing of synergy in the combination.

**Figure 6:**
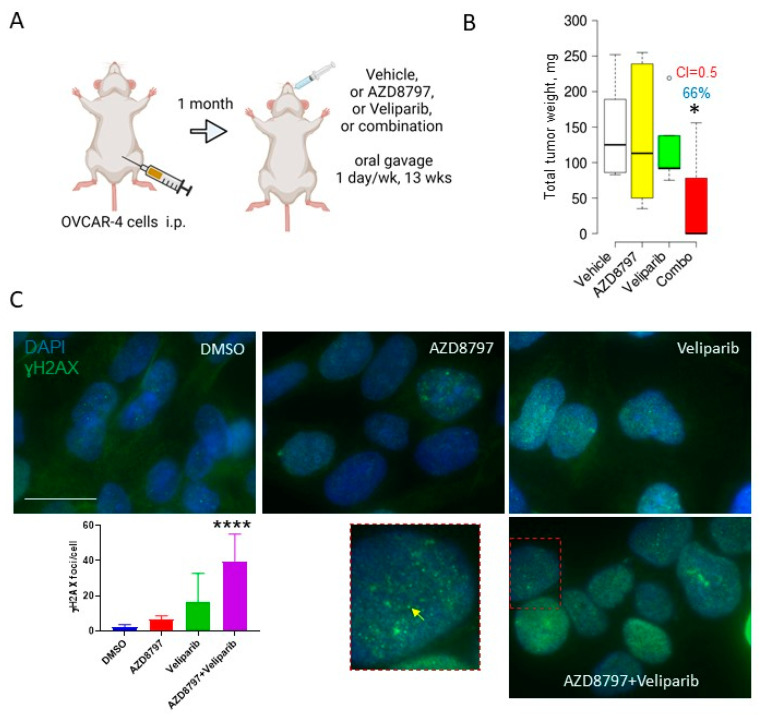
Combining veliparib and AZD8797 robustly and synergistically reduces tumor weight in the OVCAR-4 xenograft model. (**A**) The scheme represents an experimental design in which 10^6^ OVCAR-4 cells were i.p. injected into athymic nude mice (n = 10/group), allowed to form tumors for 1 month, followed by treatment with either AZD8797 (0.33 mg/mouse) or veliparib (0.33 mg/mouse) or their combination that was delivered by oral gavage once weekly for thirteen weeks, and sacrificed. The images were created in BioRender. (**B**) At the time of sacrifice, the tumor was collected, measured, plotted with BoxPlotR software (chemgrid.org), and analyzed using the Mann–Whitney U test by comparing the tumor weight in the vehicle-treated group to drug-treated groups. * *p* < 0.05. The combination index (CI) was quantified using the Bliss Independence model. Percent of tumor reduction is indicated on the graphs. (**C**) Phosphorylation of H2AX in vehicle- and drug-treated cells was examined with immunofluorescence staining. Cells were treated with DMSO, 1 µM veliparib, 5 µM AZD8797, and a combination of 1 µM veliparib and 5 µM AZD8797 for 48 h, fixed, immunostained with ɣH2AX-specific antibodies, incubated with DAPI, and imaged. Bar: 25 micron. ɣH2AX foci (indicated with a yellow arrow) were quantified using AxioVision 4.8 software in 100 random cells from three independent experiments, averaged, plotted, and statistically analyzed between the control and drug-treated groups with the Mann–Whitney U test. **** *p* < 0.0001.

Combinations of AZD8797 and veliparib demonstrated the strongest synergy in the OVCAR-4 cell line in our in vitro studies (Figure 2). To test whether AZD8797 and veliparib synergize in vivo, we set up another experiment that was designed to capture the combined effect of the two drugs. The drugs were administered once weekly for thirteen weeks. This regimen was expected to yield little or no effect from the monotherapies, as we expected that the six-day drug-free period would allow cells to recover from the drug treatments. At the same time, if the drugs synergistically affected the same pathway critical for survival and proliferation, the drug combination should have resulted in a substantial reduction in tumor cell growth. OVCAR-4 cells were inoculated i.p. and allowed to form tumors for 1 month to establish bigger lesions. AZD8797, veliparib, and their combination were administered once weekly for thirteen weeks, followed by sacrifice (Figure 6A). As expected, AZD8797 and veliparib did not affect tumor formation individually; however, their combination significantly and robustly reduced the tumor burden by 66%, and the drugs acted strongly, synergistically (Figure 6B), suggesting that the two drugs may be converging on the same pathway(s). These data mirror our in vitro findings that demonstrate a strong synergy between AZD8797 and veliparib in OVCAR-4.

We previously reported that the downregulation of CX_3_CR1 expression with gene-specific siRNAs results in the sustained inactivation of recognition and repair of dsDNA damage [23]. Hence, we examined the formation of ɣH2AX foci as a surrogate measure of dsDNA damage in cells treated with the vehicle, AZD8797, veliparib, and the combination of two drugs using immunofluorescence staining. We observed that the number of ɣH2AX foci significantly increased in the cells treated with the combination of AZD8797 and veliparib (Figure 6C), suggesting that the two drugs likely converge on the same pathway(s) regulating dsDNA repair.

## 4. Discussion

Since the introduction of PARPis for the clinical management of ovarian carcinoma, the outcomes of these therapies were used to inform research directions aiming to improve their efficacy. Several studies have investigated how targeting pathways contributing to HRD can enhance the effectiveness of PARPis in HGSOC. It has been recently reported that an orally bioavailable dual inhibitor of ATM and DNA-PKcs kinases synergized with PARP inhibitors in BRCA1/2-deficient models, including UWB1.289, a BRCA1-mutant human ovarian cancer cell line [39]. In a clinical trial, the addition of an inhibitor of ATR resensitized HR-deficient platinum-sensitive recurrent HGSOC to olaparib that previously benefited and then progressed with PARPis [40]. A combination of a CDK4/6 inhibitor palbociclib with olaparib in ovarian cancer cell models demonstrated a synergistic effect in MYC-overexpressing cells, presumably via palbociclib-induced HRD [41]. These studies suggest that rational combinations of PARP inhibitors with other drugs that converge on DNA repair pathways could benefit patients with HR-deficient and -proficient tumors.

In this study, our approach aimed to investigate whether the inhibition of CX_3_CR1, which leads to reduced HR efficiency [23], could synergize with PARPis in eliminating ovarian cancer cells. There are several potential advantages of targeting CX_3_CR1 among other potential HRD-inducing approaches. AZD8797 is an orally available investigational drug [36], and this route of administration may be preferred over intravenous or intraperitoneal infusion. The CX_3_CL1/CX_3_CR1 axis is highly specific; hence, its impairment is not expected to interfere with functions supported by other chemokines and chemokine receptors. We previously demonstrated that CX_3_CR1 was expressed in the majority of the tested patient cases; and our studies also demonstrated that the CX_3_CL1/CX_3_CR1 axis plays a role in mesothelial cell adhesion, cell proliferation, and cell migration, i.e., some of the essential functions of a metastatic cell, which is essential for blocking metastatic recolonization [26,27]. CX_3_CR1 is ubiquitous in pathological conditions, but is seldom expressed in normal tissues [42,43,44,45,46]. The disadvantages of a fast integration of this approach into the clinic include the yet unknown on-target and off-target effects of AZD8797 in the treatment of HGSOC and the tolerability of PARPi combinations with AZD8797.

Our data demonstrate that co-targeting CX_3_CR1 and PARP in in vitro models of HGSOC results in distinct outcomes as it relates to drug interaction, ranging from additive to strong synergistic. We found that co-targeting CX_3_CR1 in OVCAR-4 generated a weak to strong synergy with all four tested PARPis, olaparib, niraparib, rucaparib, and veliparib. In other cell culture models with wild-type BRCA, such as Caov-3 and ES2, the nature of the drug interaction ranged from additive to strongly synergistic. Moreover, in a model of a dysfunctional BRCA2, OVSAHO, some PARPis (olaparib and rucaparib) demonstrated an additive nature of interaction with AZD8797, while other PARPis (niraparib and veliparib) were synergistic, suggesting that a loss of BRCA2 is not the limiting factor that induces synthetic lethality for those drug combinations. Likewise, for each tested PARPi, the nature of the drug interaction with AZD8797 ranged from additive to strongly synergistic, depending on the cell line. To sum up, there was not one distinct cell line in which co-targeting CX_3_CR1 and PARP produced a strong synergy with all the tested PARPis. Likewise, there was not one PARPi that was strongly synergistic with AZD8797 in all the tested cell culture models.

Importantly, a strong synergy between AZD8797 and veliparib was observed in OVCAR-4 and Caov-3. And a strong synergy between AZD8797 and rucaparib was observed in Caov-3 and OVSAHO. Based on our data demonstrating a significant increase in the number of ɣH2AX foci in cells treated with the combination of AZD8797 and veliparib, we posit that the two drugs converge on a mechanism that regulates DNA repair.

In attempting to explain the discrepancy in the nature of the drug interaction between AZD8797 and PARPis in the tested HGSOC models, we propose that it is likely that several potential underlying mechanisms may contribute to the overall outcome, possibly to a different degree.

PARP inhibitors bind to the nicotinamide-binding site of PARPs. The efficiency of PARP1 inhibition for all four tested PARPis is very similar, with Ki values ranging between 1.4 and 5.2 nM. Similarly, olaparib, niraparib, and veliparib inhibit PARP2 at nanomolar concentrations (Ki values between 1.0 and 2.9 nM), although rucaparib is slightly less efficient at inhibiting PARP2, with a Ki value of 28 nM [47,48,49,50]. Furthermore, PARP trapping efficiency is similar for niraparib, olaparib, and rucaparib, and it is higher than that for veliparib [51]. Hence, PARPi activities against PARPs do not easily explain the differences in the observed outcomes for their combinations with CX_3_CR1.

Clinically observed adverse effects are not identical among PARP inhibitors, suggesting a potential for off-target effects [52]. Hence, studies examined the polypharmacology of PARPis and found several off-target genes differentially affected by PARPis. A study examined the effects of rucaparib, olaparib, and veliparib of a panel of kinases and found that rucaparib inhibits nine kinases with micromolar affinity, including PIM1, PIM2, PRKD2, DYRK1A, CDK1, CDK9, HIPK2, CK2, and ALK; olaparib does not inhibit any of tested kinases; and veliparib inhibits PIM1 and CDK9 [53]. In another study, it was demonstrated that niraparib and rucaparib inhibit DYRK1s, CDK16, and PIM3 at clinically achievable concentrations [52]. Hence, it is possible that the off-target effects of PARPis can contribute to the degree to which AZD8797 and PARPis synergize in the tested models of HGSOC, but more studies are needed to examine the role of all potential effectors.

In comparing some of the genetic features of the tested cell lines, all express wild-type BRCA1, and all but OVSAHO, which has a homozygous deletion of BRCA2, also express wild-type BRCA2. All tested cell lines have different mutations in TP53; OVCAR-4, Caov-3, and ES2 have mutations within the DNA-binding domain (residues 130, 136, and 241, respectively [33,54,55]), and OVSAHO carries a p53 mutation in the tetramerization domain (residue R342 [56]). Less studied is all the potential changes in the expression of other genes contributing to HRD [57,58]. A better understanding of how HRD overall and individual genes contribute to PARPi vulnerability is likely to inform the potential outcomes of the treatment.

All cell lines express similar levels of CX_3_CR1; however, it remains unclear what isoform(s) of CX_3_CR1 and what type of G proteins are expressed in each cell line. Furthermore, AZD8797 was described as an allosteric inhibitor of CX_3_CR1 [37]; however, it is not known whether it leads to the same changes in the receptor conformation and affects the downstream signaling cascades in all tested ovarian cancer cell lines similarly. Potentially, testing other available pharmacologic inhibitors of CX_3_CR1, such as JMS-17-2 [59], and using alternative targeting approaches, such as genomic downregulation via siRNAs, shRNAs, and by CRISPR/Cas9 knock-down, could be useful for a better understanding the specifics of targeting CX_3_CR1 that can impact the efficacy of PARPis.

While there is no clear pattern emerging from comparing the expression and mutation status of CX_3_CR1, BRCA1/2, and p53, there could be other genetic factors, such as off-target genes affected by either AZD8797 or PARPis, or both. AZD8797 inhibits another chemokine receptor, CXCR2, with an affinity 100-times higher than that for CX_3_CR1 [37]; however, it is not known whether it affects any other chemokine receptors, G protein-coupled receptors other than chemokine receptors, any unrelated membrane-inserted, intracellular, or extracellular proteins. Hence, it is likely that there are yet unknown factors important for HGSOC cell survival that could be synergistically affected by AZD8797 and either veliparib or rucaparib, and they are likely related to the molecular features of the tumor itself.

To expand the translational potential of our study, it is of utmost importance to determine the molecular features of the tumor that make it particularly vulnerable to the combinations of AZD8797 and either veliparib or rucaparib in the follow-up studies. Additionally, testing different doses and times of drug administration in preclinical models is essential for refining the treatment regimen and gaining a better understanding of how in vitro studies translate into the in vivo setting. Another important consideration for future studies is the importance of clinical trials to demonstrate the efficacy and tolerability of the PARPi combinations with AZD8797 in patients.

## 5. Conclusions

We hypothesized that the induction of HRD via the inhibition of CX_3_CR1 could sensitize ovarian cancer cells to PARPis. To test this premise, we combined a CX_3_CR1 inhibitor with several inhibitors of PARP and investigated the efficacy of these combinations in ovarian cancer cell lines and xenograft models. Our data indicate that most combinations result in a synergistic reduction in the clonogenic ability, while some combinations produce additive effects. A combination of the CX_3_CR1 inhibitor with veliparib was strongly synergistic in reducing clone formation in OVCAR-4 and reducing tumor burden in OVCAR-4/athymic nude mouse xenograft experiments. The data indicate that targeting CX_3_CR1 to induce HRD and sensitize to PARPis could be a potentially viable therapeutic approach, although more research is needed to further optimize the drug treatment regimen in preclinical models and to identify the molecular profile of cancers that are likely to respond to this therapeutic approach.

## Data Availability

Data is contained within the article or supplementary material.

## Supplementary Materials

The following supporting information can be downloaded at: https://www.mdpi.com/article/10.3390/cancers16223728/s1, original image of Western blot for Figure 1 and Figure 3.

## References

[1] Siegel R.L., Giaquinto A.N., Jemal A. (2024). Cancer statistics, 2024. CA Cancer J. Clin..

[2] Karnezis A.N., Cho K.R. (2017). Of mice and women—Non-ovarian origins of “ovarian” cancer. Gynecol. Oncol..

[3] Kurman R.J., Shih Ie M. (2016). The Dualistic Model of Ovarian Carcinogenesis: Revisited, Revised, and Expanded. Am. J. Pathol..

[4] Singh N., McCluggage W.G., Gilks C.B. (2017). High-grade serous carcinoma of tubo-ovarian origin: Recent developments. Histopathology.

[5] Forstner R. (2020). Early detection of ovarian cancer. Eur. Radiol..

[6] Lengyel E. (2010). Ovarian cancer development and metastasis. Am. J. Pathol..

[7] Kurnit K.C., Fleming G.F., Lengyel E. (2021). Updates and New Options in Advanced Epithelial Ovarian Cancer Treatment. Obstet. Gynecol..

[8] Ortiz M., Wabel E., Mitchell K., Horibata S. (2022). Mechanisms of chemotherapy resistance in ovarian cancer. Cancer Drug Resist..

[9] Cancer Genome Atlas Research Network (2011). Integrated genomic analyses of ovarian carcinoma. Nature.

[10] Konstantinopoulos P.A., Ceccaldi R., Shapiro G.I., D’Andrea A.D. (2015). Homologous Recombination Deficiency: Exploiting the Fundamental Vulnerability of Ovarian Cancer. Cancer Discov..

[11] Ray Chaudhuri A., Nussenzweig A. (2017). The multifaceted roles of PARP1 in DNA repair and chromatin remodelling. Nat. Rev..

[12] Miller R.E., Elyashiv O., El-Shakankery K.H., Ledermann J.A. (2022). Ovarian Cancer Therapy: Homologous Recombination Deficiency as a Predictive Biomarker of Response to PARP Inhibitors. OncoTargets Ther..

[13] Gonzalez-Martin A., Pothuri B., Vergote I., DePont Christensen R., Graybill W., Mirza M.R., McCormick C., Lorusso D., Hoskins P., Freyer G. (2019). Niraparib in Patients with Newly Diagnosed Advanced Ovarian Cancer. N. Engl. J. Med..

[14] Banerjee S., Moore K.N., Colombo N., Scambia G., Kim B.G., Oaknin A., Friedlander M., Lisyanskaya A., Floquet A., Leary A. (2021). Maintenance olaparib for patients with newly diagnosed advanced ovarian cancer and a BRCA mutation (SOLO1/GOG 3004): 5-year follow-up of a randomised, double-blind, placebo-controlled, phase 3 trial. Lancet Oncol..

[15] Pujade-Lauraine E., Ledermann J.A., Selle F., Gebski V., Penson R.T., Oza A.M., Korach J., Huzarski T., Poveda A., Pignata S. (2017). Olaparib tablets as maintenance therapy in patients with platinum-sensitive, relapsed ovarian cancer and a BRCA1/2 mutation (SOLO2/ENGOT-Ov21): A double-blind, randomised, placebo-controlled, phase 3 trial. Lancet Oncol..

[16] Mirza M.R., Monk B.J., Herrstedt J., Oza A.M., Mahner S., Redondo A., Fabbro M., Ledermann J.A., Lorusso D., Vergote I. (2016). Niraparib Maintenance Therapy in Platinum-Sensitive, Recurrent Ovarian Cancer. N. Engl. J. Med..

[17] Coleman R.L., Oza A.M., Lorusso D., Aghajanian C., Oaknin A., Dean A., Colombo N., Weberpals J.I., Clamp A., Scambia G. (2017). Rucaparib maintenance treatment for recurrent ovarian carcinoma after response to platinum therapy (ARIEL3): A randomised, double-blind, placebo-controlled, phase 3 trial. Lancet.

[18] Coleman R.L., Fleming G.F., Brady M.F., Swisher E.M., Steffensen K.D., Friedlander M., Okamoto A., Moore K.N., Efrat Ben-Baruch N., Werner T.L. (2019). Veliparib with First-Line Chemotherapy and as Maintenance Therapy in Ovarian Cancer. N. Engl. J. Med..

[19] Bhamidipati D., Haro-Silerio J.I., Yap T.A., Ngoi N. (2023). PARP inhibitors: Enhancing efficacy through rational combinations. Br. J. Cancer.

[20] Hockings H., Miller R.E. (2023). The role of PARP inhibitor combination therapy in ovarian cancer. Ther. Adv. Med. Oncol..

[21] Alvarez Secord A., O’Malley D.M., Sood A.K., Westin S.N., Liu J.F. (2021). Rationale for combination PARP inhibitor and antiangiogenic treatment in advanced epithelial ovarian cancer: A review. Gynecol. Oncol..

[22] Gomez-Roman N., Chong M.Y., Chahal S.K., Caragher S.P., Jackson M.R., Stevenson K.H., Dongre S.A., Chalmers A.J. (2020). Radiation Responses of 2D and 3D Glioblastoma Cells: A Novel, 3D-specific Radioprotective Role of VEGF/Akt Signaling through Functional Activation of NHEJ. Mol. Cancer Ther..

[23] Xie J., Gurler Main H., Sacks J.D., Muralidhar G.G., Barbolina M.V. (2018). Regulation of DNA damage repair and lipid uptake by CX3CR1 in epithelial ovarian carcinoma. Oncogenesis.

[24] Chou T.C., Talalay P. (1984). Quantitative analysis of dose-effect relationships: The combined effects of multiple drugs or enzyme inhibitors. Adv. Enzyme Regul..

[25] Zhao W., Sachsenmeier K., Zhang L., Sult E., Hollingsworth R.E., Yang H. (2014). A New Bliss Independence Model to Analyze Drug Combination Data. J. Biomol. Screen..

[26] Gurler Main H., Xie J., Muralidhar G.G., Elfituri O., Xu H., Kajdacsy-Balla A.A., Barbolina M.V. (2017). Emergent role of the fractalkine axis in dissemination of peritoneal metastasis from epithelial ovarian carcinoma. Oncogene.

[27] Kim M., Rooper L., Xie J., Kajdacsy-Balla A.A., Barbolina M.V. (2012). Fractalkine receptor CX(3)CR1 is expressed in epithelial ovarian carcinoma cells and required for motility and adhesion to peritoneal mesothelial cells. Mol. Cancer Res..

[28] Barbolina M.V. (2022). Targeting Microtubule-Associated Protein Tau in Chemotherapy-Resistant Models of High-Grade Serous Ovarian Carcinoma. Cancers.

[29] Sacks Suarez J., Gurler Main H., Muralidhar G.G., Elfituri O., Xu H.L., Kajdacsy-Balla A.A., Barbolina M.V. (2019). CD44 Regulates Formation of Spheroids and Controls Organ-Specific Metastatic Colonization in Epithelial Ovarian Carcinoma. Mol. Cancer Res..

[30] Stordal B., Timms K., Farrelly A., Gallagher D., Busschots S., Renaud M., Thery J., Williams D., Potter J., Tran T. (2013). BRCA1/2 mutation analysis in 41 ovarian cell lines reveals only one functionally deleterious BRCA1 mutation. Mol. Oncol..

[31] Meijer T.G., Martens J.W.M., Prager-van der Smissen W.J.C., Verkaik N.S., Beaufort C.M., van Herk S., Robert-Finestra T., Hoogenboezem R.M., Ruigrok-Ritstier K., Paul M.W. (2024). Functional Homologous Recombination (HR) Screening Shows the Majority of BRCA1/2-Mutant Breast and Ovarian Cancer Cell Lines Are HR-Proficient. Cancers.

[32] Domcke S., Sinha R., Levine D.A., Sander C., Schultz N. (2013). Evaluating cell lines as tumour models by comparison of genomic profiles. Nat. Commun..

[33] Concin N., Stimpfl M., Zeillinger C., Wolff U., Hefler L., Sedlak J., Leodolter S., Zeillinger R. (2003). Role of p53 in G2/M cell cycle arrest and apoptosis in response to gamma-irradiation in ovarian carcinoma cell lines. Int. J. Oncol..

[34] Etemadmoghadam D., George J., Cowin P.A., Cullinane C., Kansara M., Australian Ovarian Cancer Study G., Gorringe K.L., Smyth G.K., Bowtell D.D. (2010). Amplicon-dependent CCNE1 expression is critical for clonogenic survival after cisplatin treatment and is correlated with 20q11 gain in ovarian cancer. PLoS ONE.

[35] Stock E., Schab A., Oplt A., Noia H., Lomonosova E., Bruce S., Khabele D., Kuroki L., Hagemann A., McCourt C. (2021). Increasing sensitivity to olaparib through inhibition of discoidin domain receptor 2 (DDR2) in homologous-recombination proficient ovarian cancer models. Gynecol. Oncol..

[36] Karlstrom S., Nordvall G., Sohn D., Hettman A., Turek D., Ahlin K., Kers A., Claesson M., Slivo C., Lo-Alfredsson Y. (2013). Substituted 7-amino-5-thio-thiazolo[4,5-d]pyrimidines as potent and selective antagonists of the fractalkine receptor (CX3CR1). J. Med. Chem..

[37] Cederblad L., Rosengren B., Ryberg E., Hermansson N.O. (2016). AZD8797 is an allosteric non-competitive modulator of the human CX3CR1 receptor. Biochem. J..

[38] Adam R.A., Adam Y.G. (2004). Malignant ascites: Past, present, and future. J. Am. Coll. Surg..

[39] Gilmer T.M., Lai C.H., Guo K., Deland K., Ashcraft K.A., Stewart A.E., Wang Y., Fu J., Wood K.C., Kirsch D.G. (2024). A Novel Dual ATM/DNA-PK Inhibitor, XRD-0394, Potently Radiosensitizes and Potentiates PARP and Topoisomerase I Inhibitors. Mol. Cancer Ther..

[40] Wethington S.L., Shah P.D., Martin L., Tanyi J.L., Latif N., Morgan M., Torigian D.A., Rodriguez D., Smith S.A., Dean E. (2023). Combination ATR (ceralasertib) and PARP (olaparib) Inhibitor (CAPRI) Trial in Acquired PARP Inhibitor-Resistant Homologous Recombination-Deficient Ovarian Cancer. Clin. Cancer Res..

[41] Yi J., Liu C., Tao Z., Wang M., Jia Y., Sang X., Shen L., Xue Y., Jiang K., Luo F. (2019). MYC status as a determinant of synergistic response to Olaparib and Palbociclib in ovarian cancer. EBioMedicine.

[42] D’Haese J.G., Demir I.E., Friess H., Ceyhan G.O. (2010). Fractalkine/CX3CR1: Why a single chemokine-receptor duo bears a major and unique therapeutic potential. Expert Opin. Ther. Targets.

[43] D’Haese J.G., Friess H., Ceyhan G.O. (2012). Therapeutic potential of the chemokine-receptor duo fractalkine/CX3CR1: An update. Expert Opin. Ther. Targets.

[44] Medina-Contreras O., Geem D., Laur O., Williams I.R., Lira S.A., Nusrat A., Parkos C.A., Denning T.L. (2011). CX3CR1 regulates intestinal macrophage homeostasis, bacterial translocation, and colitogenic Th17 responses in mice. J. Clin. Investig..

[45] Rooper L., Gurler H., Kajdacsy-Balla A.A., Barbolina M.V. (2013). Fractalkine receptor is expressed in mature ovarian teratomas and required for epidermal lineage differentiation. J. Ovarian Res..

[46] Zhang Q., Shimoya K., Temma K., Kimura T., Tsujie T., Shioji M., Wasada K., Fukui O., Hayashi S., Kanagawa T. (2004). Expression of fractalkine in the Fallopian tube and of CX3CR1 in sperm. Hum. Reprod..

[47] Menear K.A., Adcock C., Boulter R., Cockcroft X.L., Copsey L., Cranston A., Dillon K.J., Drzewiecki J., Garman S., Gomez S. (2008). 4-[3-(4-cyclopropanecarbonylpiperazine-1-carbonyl)-4-fluorobenzyl]-2H-phthalazin-1-one: A novel bioavailable inhibitor of poly(ADP-ribose) polymerase-1. J. Med. Chem..

[48] Thomas H.D., Calabrese C.R., Batey M.A., Canan S., Hostomsky Z., Kyle S., Maegley K.A., Newell D.R., Skalitzky D., Wang L.Z. (2007). Preclinical selection of a novel poly(ADP-ribose) polymerase inhibitor for clinical trial. Mol. Cancer Ther..

[49] Donawho C.K., Luo Y., Luo Y., Penning T.D., Bauch J.L., Bouska J.J., Bontcheva-Diaz V.D., Cox B.F., DeWeese T.L., Dillehay L.E. (2007). ABT-888, an orally active poly(ADP-ribose) polymerase inhibitor that potentiates DNA-damaging agents in preclinical tumor models. Clin. Cancer Res..

[50] Jones P., Altamura S., Boueres J., Ferrigno F., Fonsi M., Giomini C., Lamartina S., Monteagudo E., Ontoria J.M., Orsale M.V. (2009). Discovery of 2-4-[(3S)-piperidin-3-yl]phenyl-2H-indazole-7-carboxamide (MK-4827): A novel oral poly(ADP-ribose)polymerase (PARP) inhibitor efficacious in BRCA-1 and -2 mutant tumors. J. Med. Chem..

[51] Murai J., Pommier Y. (2015). O6.1—Classification of PARP inhibitors based on PARP trapping and catalytic inhibition, and rationale for combinations. Ann. Oncol..

[52] Antolin A.A., Ameratunga M., Banerji U., Clarke P.A., Workman P., Al-Lazikani B. (2020). The kinase polypharmacology landscape of clinical PARP inhibitors. Sci. Rep..

[53] Antolin A.A., Mestres J. (2014). Linking off-target kinase pharmacology to the differential cellular effects observed among PARP inhibitors. Oncotarget.

[54] Ikediobi O.N., Davies H., Bignell G., Edkins S., Stevens C., O’Meara S., Santarius T., Avis T., Barthorpe S., Brackenbury L. (2006). Mutation analysis of 24 known cancer genes in the NCI-60 cell line set. Mol. Cancer Ther..

[55] Yaginuma Y., Westphal H. (1992). Abnormal structure and expression of the p53 gene in human ovarian carcinoma cell lines. Cancer Res..

[56] Elias K.M., Emori M.M., Papp E., MacDuffie E., Konecny G.E., Velculescu V.E., Drapkin R. (2015). Beyond genomics: Critical evaluation of cell line utility for ovarian cancer research. Gynecol. Oncol..

[57] Ngoi N.Y.L., Tan D.S.P. (2021). The role of homologous recombination deficiency testing in ovarian cancer and its clinical implications: Do we need it?. ESMO Open.

[58] Mangogna A., Munari G., Pepe F., Maffii E., Giampaolino P., Ricci G., Fassan M., Malapelle U., Biffi S. (2023). Homologous Recombination Deficiency in Ovarian Cancer: From the Biological Rationale to Current Diagnostic Approaches. J. Pers. Med..

[59] Shen F., Zhang Y., Jernigan D.L., Feng X., Yan J., Garcia F.U., Meucci O., Salvino J.M., Fatatis A. (2016). Novel Small-molecule CX3CR1 Antagonist Impairs Metastatic Seeding and Colonization of Breast Cancer Cells. Mol. Cancer Res..

