# Supplementary figures and images for: Dual Targeting of CX3CR1 and PARP in Models of High-Grade Serous Ovarian Carcinoma
Source: Cancers (Basel). 2024 Nov 5;16(22):3728. doi: 10.3390/cancers16223728 (PMC11591600; doi:10.3390/cancers16223728)

1<sup>st</sup> set

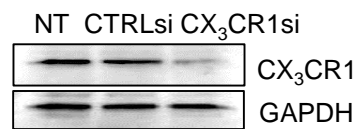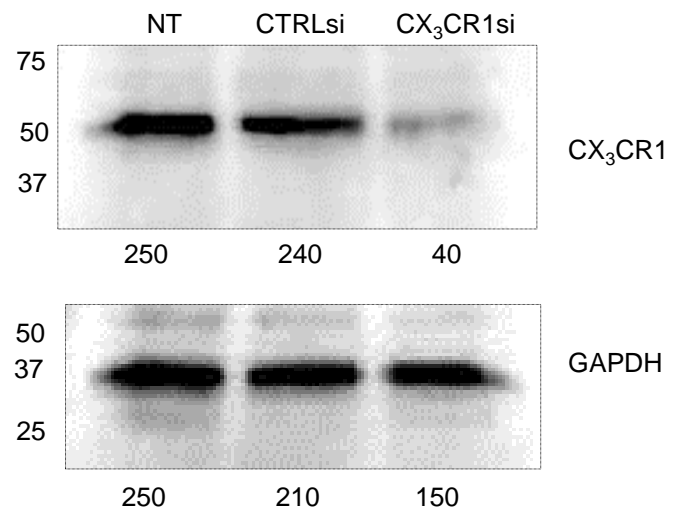

2<sup>nd</sup> set

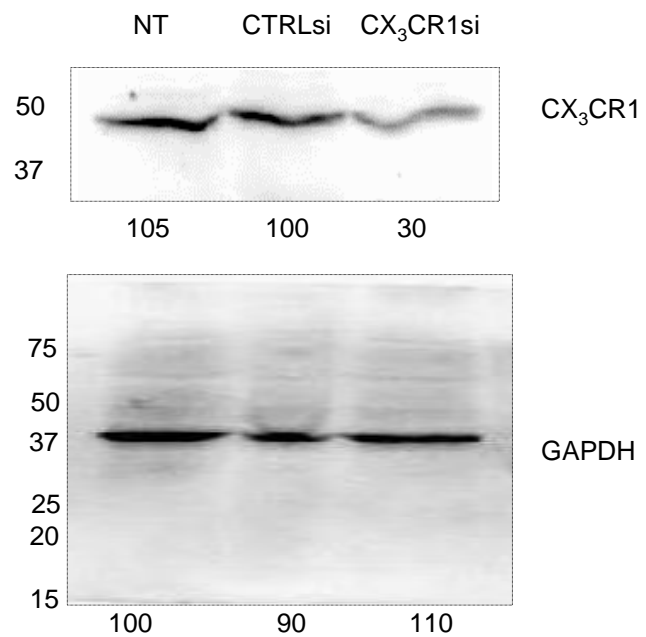

3<sup>rd</sup> set

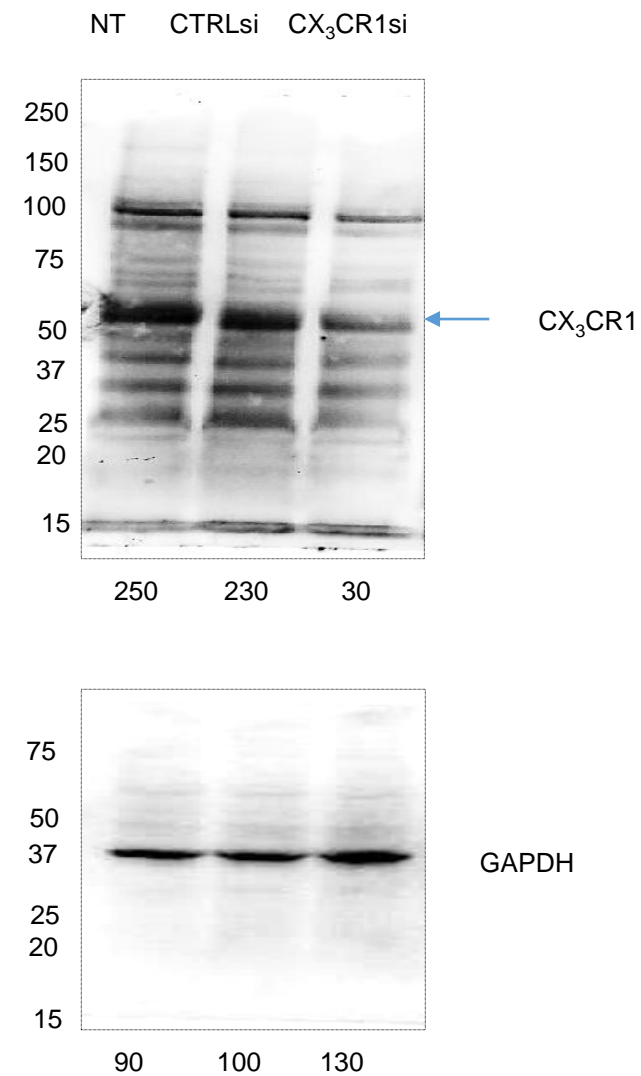

original image of Western blot for Figure 3

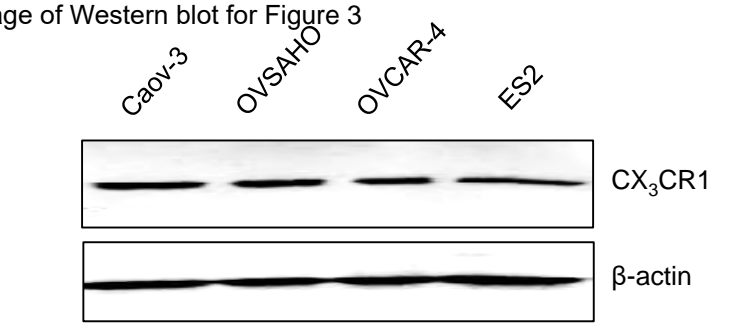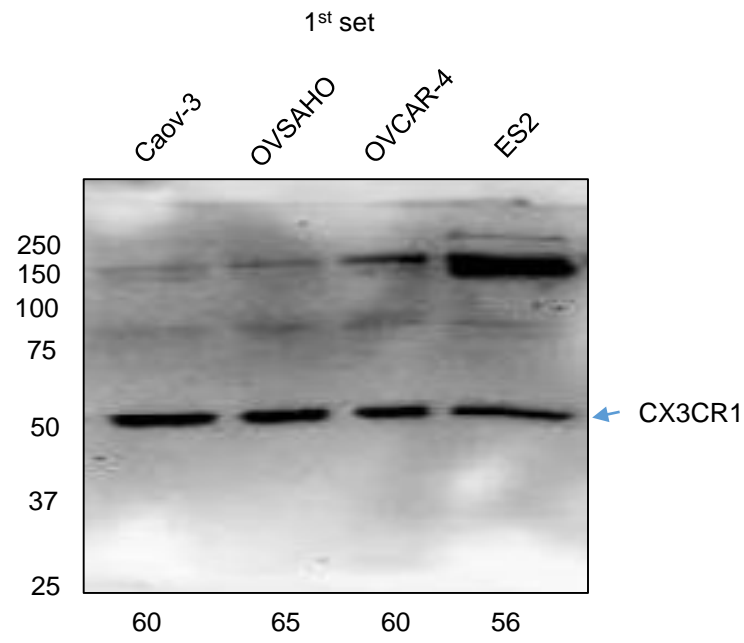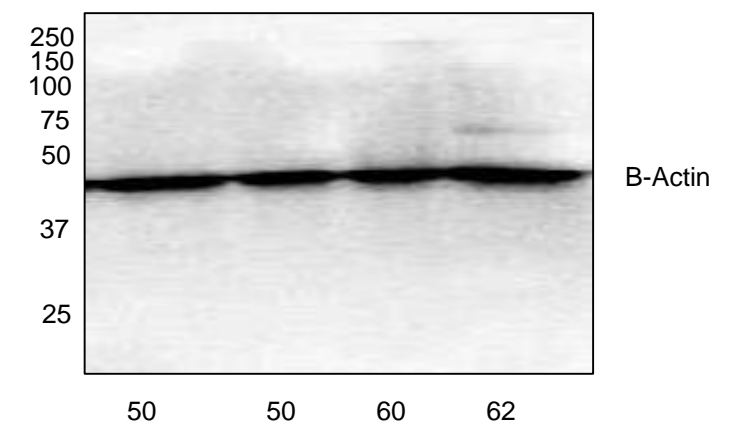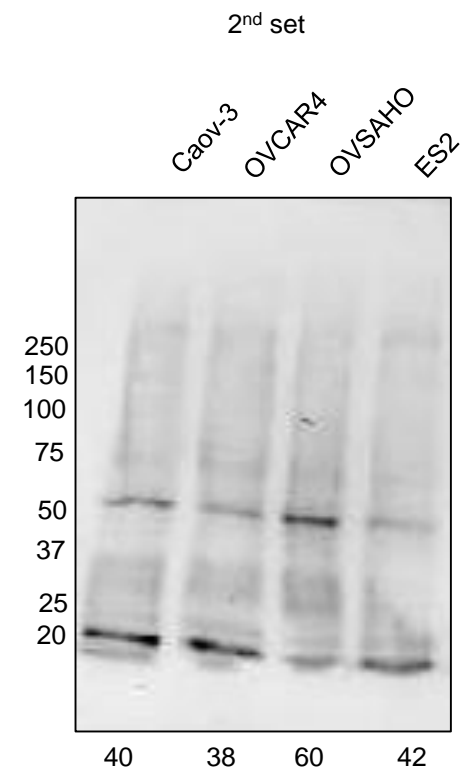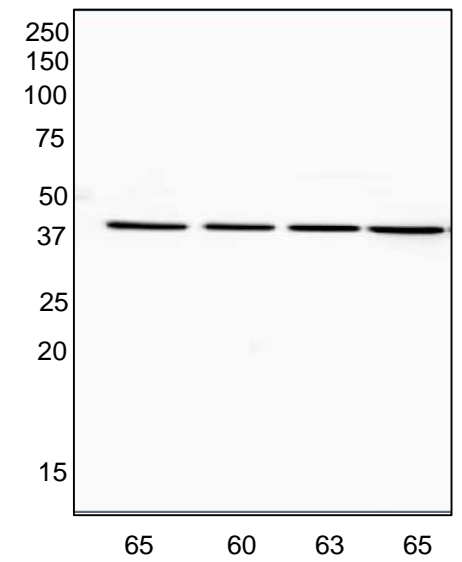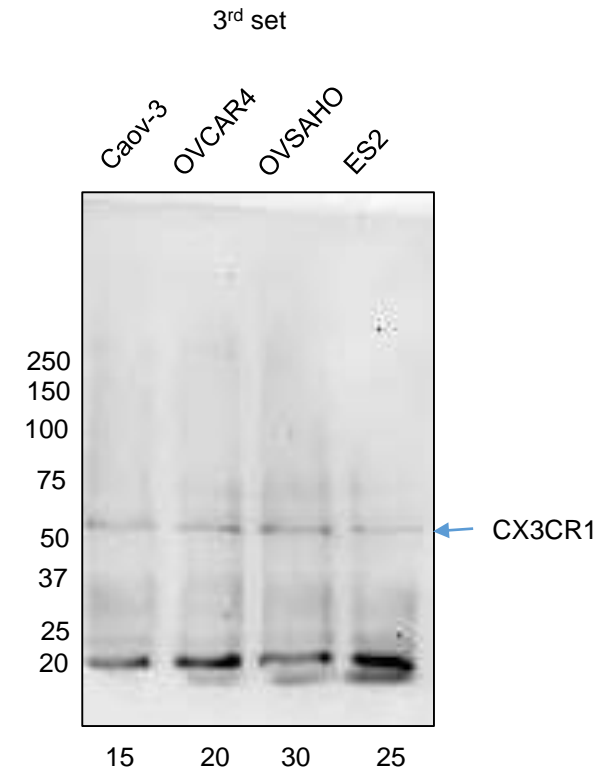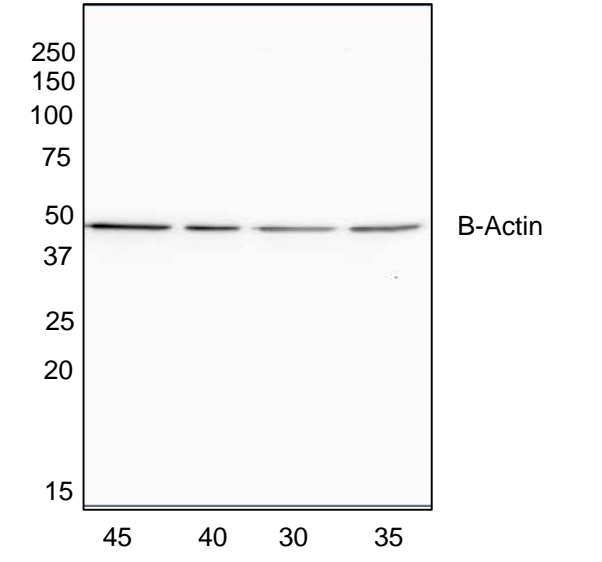

Supplement: Supplementary file 1 [file cancers-16-03728-s001.zip › cancers-3279510-supplementary.pdf]
